# Clinical and environmental wastewater-based bacteriophage surveillance for high-impact diarrheal diseases, including cholera, in Bangladesh

**DOI:** 10.1128/mbio.02654-25

**Published:** 2025-12-09

**Authors:** Marjahan Akhtar, Md. Ariful Amin, Subah Nuzhat Hussain, Nazia Nazrul Nafsi, Nasrin Parvin, Farhana Khanam, Md. Taufiqul Islam, Md. Amirul Islam Bhuiyan, Rahima Afroz, Md. Golam Firoj, Fahima Chowdhury, Ashraful Islam Khan, Mohammad Jubair, Edward T. Ryan, B. Jesse Shapiro, Nicholas R. Thomson, Eric J. Nelson, Md. Mustafizur Rahman, Yasmin Ara Begum, Taufiqur Rahman Bhuiyan, Firdausi Qadri

**Affiliations:** 1 Infectious Diseases Division, Bangladesh (icddr,b), International Centre for Diarrhoeal Disease Research 56291, Dhaka, Bangladesh; 2 Division of Infectious Diseases, Massachusetts General Hospital, Boston, Massachusetts, USA; 3 Department of Medicine, Harvard Medical School, Boston, Massachusetts, USA; 4 Department of Immunology and Infectious Diseases, Harvard T.H. Chan School of Public Health, Boston, Massachusetts, USA; 5 Department of Microbiology and Immunology, McGill University 5620, Montreal, Canada; 6 Parasites and Microbes, Wellcome Sanger Institute, Wellcome Genome Campus, Hinxton, Cambridgeshire, United Kingdom; 7 Department of Pathogen Molecular Biology, Faculty of Infectious and Tropical Diseases, London School of Hygiene & Tropical Medicine, London, United Kingdom; 8 Departments of Pediatrics and Environmental and Global Health, University of Florida, Gainesville, Florida, USA; Universiteit Gent, Gent, Belgium

**Keywords:** phages, cholera, ETEC, *Shigella*, *Salmonella*, wastewater

## Abstract

Bacteriophages (phages) likely play a critical role in modulating transmission dynamics of diarrheal pathogens. This study investigated the role of phages in modulating the prevalence and seasonal patterns of major diarrheal pathogens, *Vibrio cholerae* O1 (VCO1), enterotoxigenic *Escherichia coli* (ETEC), *Shigella* spp., and *Salmonella* spp. in diarrheal patients and environmental wastewater specimens collected from six different sites in Dhaka, Bangladesh, in 2024. VCO1, ETEC, *Shigella*, and *Salmonella* were detected in 10.1%, 7.8%, 1.7%, and 2.4% of diarrheal specimens, respectively. In contrast, phages targeting these pathogens were more frequently isolated, with detection rates of 20% for VCO1, 30% for ETEC, 57% for *Shigella*, and 9.2% for *Salmonella*-specific phages. Adults showed a significantly higher burden of VCO1 and corresponding phages compared with children <5 years (*P* < 0.001). Seasonal analysis revealed significant correlations between VCO1 (37.3%) and corresponding phages (57.6%) peaking in late September in both clinical (*r* = 0.53, *P* < 0.0001) and environmental wastewater specimens (*r* = 0.65, *P* < 0.001). The highest correlation (r = 0.68) was found between the increased rate of wastewater phages in the preceding week and a rise in cholera cases in the following week. ETEC and ETEC phages isolated from wastewater also showed strong correlations (*r* = 0.65, *P* < 0.001). Cross-specificity analysis demonstrated that VCO1 phages were highly specific to their targets, whereas ETEC and *Shigella* phages exhibited broader host ranges, with some *Shigella* phages capable of infecting ETEC and *Salmonella* spp. Overall, these findings support the hypothesis that *Vibrio* phages could serve as an alternative or complementary tool for cholera surveillance.

**IMPORTANCE:**

Understanding the dynamics between phages and their bacterial hosts is critical for elucidating disease burden; however, their potential for surveillance remains underexplored. To our knowledge, this is the first study that longitudinally investigated major diarrheal pathogens and their phages in both clinical and environmental sources to assess the potential of bacteriophages as a tool to improve diarrheal surveillance. The high frequency of phages compared to the host bacterial counterparts suggests a valuable, yet underutilized, role for phages in surveillance systems. Strong seasonal alignment between *V. cholerae* O1 and its phages, both peaking in late September, suggests that phage dynamics may reflect pathogen transmission. These preliminary observations raise the possibility that wastewater-derived *Vibrio* phages could function as early indicators of cholera burden. Future research should aim to explore the complex and poorly understood interactions between phages and their bacterial hosts, particularly how these dynamics shape pathogen populations in endemic settings.

## INTRODUCTION

Diarrheal diseases remain a leading cause of morbidity and mortality in low- and middle-income countries (LMICs) (1). In Bangladesh, poor sanitation, high population density, and natural disasters contribute to the widespread prevalence of waterborne diseases like diarrhea. The major bacterial pathogens responsible for substantial diarrheal burden in Bangladesh include *V. cholerae* O1, enterotoxigenic *Escherichia coli* (ETEC), *Shigella* spp., and *Salmonella* spp. (2). Continuous surveillance of these high-impact pathogens is important for early outbreak detection, monitoring levels of antimicrobial resistance, guiding vaccine strategies, and identifying seasonal trends that in turn inform public health policies and therapeutics. Traditional disease surveillance tools have primarily focused on the detection of these disease-causing pathogens in both clinical and environmental sources (3–5). However, in resource-limited settings, certain environmental challenges like poor water and sanitation infrastructure and the high costs of laboratory diagnostics can hinder effective disease surveillance. Developing a low-cost, accurate, scalable approach could enhance both clinical and environmental surveillance systems. In this context, using bacteriophages as epidemiological markers to track and monitor specific diarrheal pathogens may offer an alternative or adjunctive option. Virulent bacteriophages (phages) are viruses that infect and subsequently lyse susceptible bacterial hosts. They play an important role in bacterial evolution by serving as natural biocontrol agents as well as vectors facilitating the spread of virulence factors and antibiotic resistance genes through horizontal gene transfer (6, 7). Despite the recognized significance of bacteriophages in bacterial ecology, there is a lack of evidence in the modern era that systematic surveillance programs incorporating phage monitoring alongside traditional pathogen tracking are efficacious.

Understanding environmental and biological factors that influence the seasonal variations of important diarrheal pathogens is critical for improving disease surveillance and developing control strategies. Although several environmental factors, such as temperature, rainfall, and water salinity, have been implicated in shaping the seasonal dynamics of diarrheal pathogens (8, 9), the role of bacteriophages in modulating pathogen transmission remains less studied. In Bangladesh, where cholera or ETEC-associated diarrhea and shigellosis remain endemic, the interactions between bacterial pathogens and their specific bacteriophages could be key to understanding disease seasonality and outbreak dynamics. Studies suggest that environmental bacteriophage populations fluctuate seasonally and may influence the persistence and transmission of diarrheal pathogens in both aquatic environments and human hosts (10, 11). A recent study on *Salmonella* Typhi demonstrated that an increased typhoid burden correlated with a rise in specific bacteriophages in environmental water in that area (12). This finding suggests that bacteriophages could serve as a potential tool for rapid environmental surveillance to assess the risk of typhoid fever and other diseases in the community. In addition, the presence of vibrio phages was shown to hamper cholera diagnostics in the diarrheal patients (13). However, there is limited evidence linking the seasonal prevalence of pathogen-specific bacteriophages with clinical diarrheal cases in endemic settings.

In this study, we aimed to investigate the potential correlations between the seasonal patterns of *V. cholerae*, ETEC, and *Shigella* and *Salmonella*-associated diarrhea and the prevalence of pathogen-specific bacteriophages in diarrheal specimens, as well as environmental wastewater specimens collected from different sewage sources in Dhaka city, Bangladesh. By analyzing diarrheal cases from a surveillance program in Bangladesh, we sought to determine whether fluctuations in bacteriophage populations correspond to the seasonal variations of these bacterial pathogens.

## MATERIALS AND METHODS

### Clinical specimens

In Bangladesh, icddr,b Dhaka hospital runs a 2% diarrheal disease surveillance in which every 50th patient admitted to the hospital is enrolled for surveillance purposes. We used this surveillance system to analyze both diarrheal pathogens and corresponding phages from the fecal specimens collected weekly from the enrolled specimens from January to December 2024. A total of 3,123 fecal specimens were collected in 2024, including 915 (29%) adults and 2,208 (71%) children. Demographic information of the study participants was shown in Table 1.

**TABLE 1:** Demographic characteristics of diarrheal patients

Parameter	Diarrheal patients (*n* = 3,123)
Age distribution, *n* (%)	
>5 years	2,055 (65.8)
5–17 years	153 (4.89)
>18 years	915 (29.29)
Sex, *n* (%)	
Male	1,761 (56.38)
Female	1,362 (43.61)
Dehydration status, *n* (%)	
Severe	443 (14.19)
Mild	652 (20.87)
None	2,028 (64.94)
Month-wise patient enrollment, *n* (%)	
January	235 (7.52)
February	284 (9.09)
March	298 (9.54)
April	216 (6.91)
May	280 (8.97)
June	279 (8.93)
July	226 (7.23)
August	150 (4.80)
September	215 (6.88)
October	257 (8.22)
November	267 (8.55)
December	416 (13.32)

### Environmental wastewater

Environmental wastewater from sewage sources (*n* = 144) was collected from six distinct locations in Dhaka city once every week from July to December 2024. Those sites were selected by the study team by mapping the location and direction of sewage flow. Three of the locations were from the Dakkhinkhan area, and three were from the Mirpur area. Every week, wastewater samples were collected before 9 am in a sterile bottle (Nalgene) and transported to the laboratory. In the laboratory, wastewater samples were settled first and filtered using a 0.22 µm Durapore Membrane filter paper (Merck, Germany). Although the filtrates were tested for the presence of bacteriophages, the residues on the filter papers were used for the detection of the bacterial strains.

### Identification of diarrheal pathogens

Diarrheal fecal specimens were used to isolate *V. cholerae* O1, ETEC, *Salmonella*, and *Shigella* spp. (14–16). For the detection of *V. cholerae*, fecal specimens were streaked onto Tellurite Taurocholate Gelatin Agar (TTGA) and then incubated overnight at 37°C. An agglutination assay utilizing specific monoclonal antibodies was used to detect *V. cholerae* O1-Ogawa and Inaba serotypes. ETEC was determined using a conventional PCR method targeting heat-labile (LT) and heat-stable (ST) enterotoxins as described previously (3, 15). For the detection of *Shigella* and *Salmonella* spp., biochemical and serological tests were carried out (16).

### Host bacterial strains

To screen for bacteriophages, we used 28 different strains as bacterial hosts, including *V. cholerae,* ETEC, *Shigella*, and *Salmonella* spp. (Table S1). For *V. cholerae,* phages were isolated using two different *V. cholerae* O1 serotypes: Ogawa and Inaba. In addition, we used a recombinant *V. cholerae* O1 strain (AC-6169, *V. cholerae* E7946 ΔRS1-CTXphage-TLC, ΔK139-attB, wbeL-A111G, A114G, manAA216G, A219G, A609G, A612G; a generous gift from Dr. Andrew Camilli at Tufts University, care of Drs. M. Alam (icddr,b) and E. Nelson (University of Florida). To identify ETEC-specific phages, we utilized 12 different ETEC strains without or with known colonization factors (CFA/I, CS1+CS3+CS21, CS2+CS3+CS21, CS5+CS6, CS4+CS6, CS6+CS8, CS7+, CS12+, CS14+, CS17+, LT+CF−, and ST+CF− ETEC strains). *Shigella*-specific phages were screened against four different *Shigella* spp.: *Shigella flexneri*, *Shigella sonnei*, *Shigella boydii*, and *Shigella dysenteriae*. Similarly, *Salmonella* phages were identified against *Salmonella enterica* serovars Typhi, Paratyphi A, and non-typhoidal *Salmonella* strains, including *S. enterica* group B and group C1. Most of the host bacterial strains (*n* = 19) were characterized using whole genome sequencing as described in the section below. An antibiogram assay was performed for the determination of antibiotic resistance as described previously (17), and the results are shown in Table S1.

### Whole genome sequencing (WGS) and bioinformatics analysis

Whole genome sequencing (WGS) was performed in the host bacterial strains. After overnight culture in Luria broth, DNA extraction was carried out using the Qiagen DNeasy Blood & Tissue Kit. The extracted DNA’s quality was evaluated for suitability for subsequent WGS using both Nanodrop (Thermo Fisher Scientific) and Qubit (Thermo Fisher Scientific) measurements. A Nanodrop spectrophotometer was used to assess DNA purity, where a 260/280 reading of 1.8 indicated high quality and minimal contamination. DNA quantification was performed using a Qubit 4.0 Fluorometer, yielding a good concentration. Library preparation was performed using the Illumina DNA Prep Reagent Kit and epMotion 5075. Prepared DNA libraries were sequenced using the Illumina NextSeq 2000 platform. The quality of these raw sequencing reads was assessed using FastQC (v0.12.1). Trimming was done to remove any adapters and low-quality sequences, employing tools such as BBDuk (v39.01) and Fastp (v0.23.4). The high-quality, trimmed reads were then ready for the subsequent analysis steps. High-quality, trimmed paired-end reads were assembled *de novo* into contigs using the SPAdes genome assembler (v4.1.0). The reads were screened for the presence of antimicrobial resistance (AMR) genes using Ariba (v2.14.6). Ariba was run against the Comprehensive Antibiotic Resistance Database (CARD) to identify known AMR genes and their associated resistance mechanisms. AMR data are shown in Table S2. To identify putative bacteriophage defense systems within the assembled genomes, the contigs were analyzed using DefenceFinder (v2.0.0). Phage resistance genes are shown in the Table S3. To predict the presence of virulence genes linked to ETEC, specifically to toxins (LTh, STh, and STp) and colonization factors, the ETEC virulence and CF database employed in this study was sourced from Astrid von Mentzer’s repository, accessible at https://github.com/avonm/.

### Identification and quantification of phages

Phages against ETEC, *V. cholerae, Shigella*, and *Salmonella* spp. were screened using a plaque assay (18, 19). Host bacterial strains were grown in Luria broth at 37°C with shaking (200 rpm) until mid-log phase (OD600 = 0.4–0.6). Bacterial lawns were prepared by spreading 1 mL of culture onto Luria agar plates and drying under aseptic conditions. This drying step ensured that phage samples were not overlapped upon spotting, allowing for clear formation of plaques. For stool specimens, approximately 100 µL of the liquid stool was diluted with PBS (1:10 dilution) and mixed with 50 µL of chloroform (Honeywell, Germany) prior to centrifugation (5,000 × *g* for 30 min). After centrifugation, 10 µL of the stool supernatants were spotted onto host bacterial lawns. Plates were incubated overnight at 37°C for clear plaque formation. For wastewater specimens, samples were settled first and filtered using a 0.22 µm Durapore Membrane filter paper (Merck, Germany). Water filtrates were enriched by co-incubation with bacterial hosts overnight at 37°C. Chloroform was added to the enriched water filtrate at a 1:5 ratio to remove host bacteria by centrifuging at 12,000 × *g* for 10 min. Enriched filtrates were then diluted 1:10 with PBS, and 10 µL aliquots of the diluted specimens were spotted onto host lawns. Plates were incubated at 37°C overnight. The appearance of clear, circular plaques indicated lytic phage activity. For confirmation, representative plaques were picked for further propagation and purification.

For quantitative assessment of plaque formation, we evaluated dose response effects of phages isolated from clinical sources (*n* = 5) and environmental wastewater sources (*n* = 5) on plaque formation and *V. cholerae* O1 growth (Fig. S3). Phage concentrations were measured using the soft agar assay. Briefly, 100 µL of serially diluted phage lysate and 100 µL of log-phase *V. cholerae* (OD₆₀₀ =0.4) culture were mixed with 4 mL of liquid soft agar and overlaid onto petri dish plates. Plates were incubated at 37°C overnight, after which plaques were counted, and concentrations were expressed as plaque-forming units per milliliter (PFU/mL). For dose response analysis, phages (10^6^ PFU/mL) were serially diluted (5-fold) to prepare 2 × 10⁵, 4 × 10⁴, 8 × 10³, 1.6 × 10², 3.2 × 10¹, and 64 PFU/mL. Each dilution was tested by soft agar assay as described above, and plaque counts were recorded. For semiconfluent and confluent lysis, we estimated approximately 5,000–10,000 plaques per plate. To determine the effect of phage dose on bacterial growth, each phage concentration was mixed with *V. cholerae* O1 growth in 96-well tissue culture plates (CELLTREAT, USA). Plates were incubated at 37°C for 3 h. Bacterial growth was measured at OD₆₀₀ using a microplate reader (EON, BioTek).

### Phage purification and cross-specificity determination

Phages identified in the plaque assay were subsequently isolated and purified to obtain single-phage populations. Single plaques were picked, diluted in PBS, and re-spotted on fresh bacterial lawns. This process was repeated at least four times to ensure the purity of the isolated phages. Purified phage lysates were stored at 4°C for short-term use and at −80°C with 50% glycerol for long-term preservation.

### Meteorological data

We collected meteorological parameters, including minimum and maximum temperature (°C) and rainfall (precipitation mm/day) of Dhaka city, Bangladesh, for the whole study period. Data were obtained from the Bangladesh Water Development Board and the Giovanni Earth data archives of the National Aeronautics and Space Administration (https://power.larc.nasa.gov/).

### Statistical analysis

Descriptive statistics for all the demographic and clinical characteristics are presented as frequencies and percentages. The χ^2^ test was used to measure the difference between the burden of diarrheal pathogens and corresponding phages in different age groups of patients. The Spearman correlation test was used to evaluate seasonal patterns of weekly isolated bacteria and phages. We performed time-lagged cross-correlation analysis across weekly lags (0–5 subsequent weeks) to investigate the association between the proportion of phages and pathogen, as well as the association between climate change (rainfall and temperature with pathogen or phages) in the subsequent weeks. All statistical tests have been conducted at the 5% level of significance. Data were analyzed by using Excel, GraphPad Prism (version 6.0), and R (version 4.4.2).

## RESULTS

### Diarrheal pathogens and phages

Among the 3,123 diarrheal specimens (fecal and rectal swabs) tested, *V. cholerae* O1 was detected in 314 (10.1%), ETEC in 244 (7.8%), and *Shigella* spp. in 52 (1.7%) of stool specimens, whereas *Salmonella* spp. was identified in 2.4% of specimens (Table 2). The burden of bacteriophages was significantly higher than that of their corresponding diarrheal pathogens in the fecal specimens (Table 2). Among the diarrheal specimens tested for phages (*n* = 1,068), 20% were positive for *V. cholerae* O1-specific phages, 30.8% for ETEC phages, 47.1% for *Shigella* phages, and 5.2% for *Salmonella* phages. Phages isolated on *V. cholerae* O1 hosts could infect both Ogawa and Inaba serotypes. The most abundant ETEC phages were those targeting CS4+CS6, CS1+CS3+CS21, CS2+CS3+CS21, CS12, CS7, CS17, and CS14. Although ETEC strains carrying CFA/I and CS5 + CS6 are the most prevalent types circulating in Bangladesh, phages specific to these strains were less frequently detected. Among the isolated *Shigella* phages, those targeting *Shigella sonnei* were the most abundant (40%), followed by *Shigella flexneri* (27%), *Shigella boydii* (13%), and *Shigella dysenteriae* (13%)-specific phages. *Salmonella* phages (9.2%) in diarrheal specimens were primarily detected against *Salmonella* Paratyphi A (34 out of 56 *Salmonella* phages), followed by non-typhoidal *Salmonella*, including *Salmonella* serogroups B and C1 and *Salmonella* Typhi (Table 2).

**TABLE 2:** Isolation rate of *V. cholerae* O1, ETEC, and *Shigella* bacteria and corresponding phages isolated from diarrheal patients

Parameter	Bacterial pathogens, *n* (%, 95% CI)	Phages, *n* (%, 95% CI)	*P* value^*a*^
Number of tested specimens	3,123	1,068	
*V. cholerae* O1	314 (10.1, 9.1–11.1)	215 (20.1, 17.8–22.7)	<0.0001
ETEC	244 (7.8, 6.9–8.8)	329 (30.8, 28.0–33.7)	<0.0001
*Shigella* spp.	52 (1.6, 1.2–2.2)	503 (47.1, 44.1–50.1)	<0.0001
*Salmonella* spp.	76 (2.4, 1.9–3.0)	55 (5.15, 3.9–6.6)	<0.0001

^*a*^ Proportion test was performed for statistical analysis.

### Temporal dynamics of *V. cholerae* O1 and phages in clinical and environmental sources

**Fig 1:**
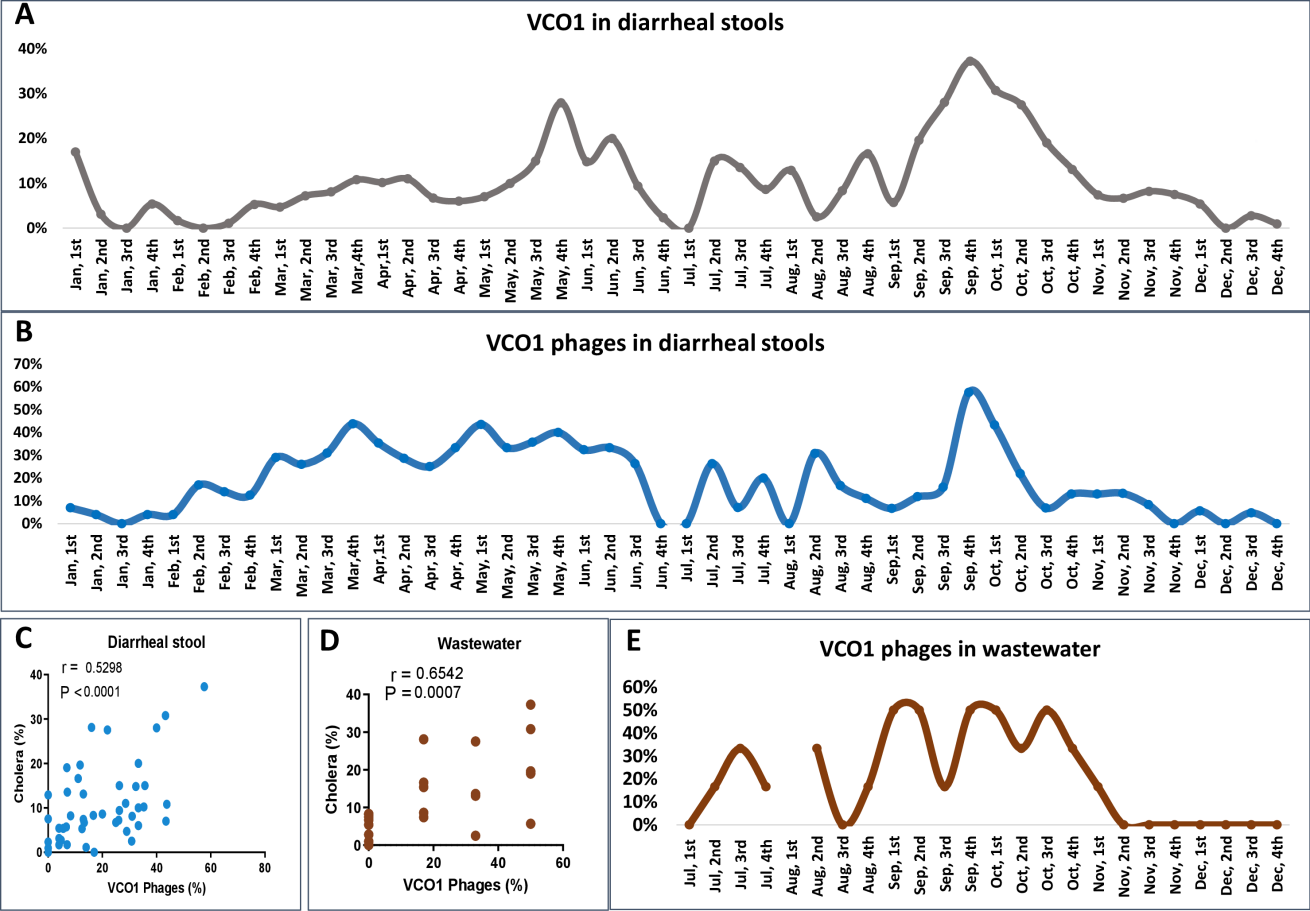
Temporal dynamics of *V. cholerae* O1 (VCO1) and corresponding phages in clinical and environmental specimens. (**A**) Weekly isolation rate of VCO1 in diarrheal specimens (*n* = 3,123) collected between January and December 2024. (**B**) Weekly isolation rate of VCO1 phages from diarrheal specimens (*n* = 1,068) from January to December 2024. (**C**) Correlation between weekly percentage of cholera cases and phages isolated from diarrheal stools. (**D**) Correlation between weekly percentage of cholera cases and phages isolated from environmental wastewater specimens (**E**) Weekly isolation rate of VCO1 phages in environmental wastewater samples collected between July and December 2024. Correlation analyses were performed using the Spearman test.

We analyzed the prevalence of *V. cholerae* O1 and its associated phages in diarrheal stools and wastewater sources over time to investigate their seasonal dynamics. In diarrheal specimens, the proportion of *V. cholerae* O1-positive samples varied throughout the year (Fig. 1A). The prevalence remained low during the earlier months but exhibited a gradual increase starting in May, declined in the first week of July, and remained low until mid-August. In late September, the cholera detection rate increased to its highest level (37.3%) before declining toward the end of the year. Similarly, phages targeting *V. cholerae* O1 in diarrheal stools followed similar fluctuating patterns of *V. cholerae* O1 prevalence (Fig. 1B). Phage detection frequencies gradually increased from the beginning of the year, with peaks corresponding to late summer (May–June) and early autumn months. Notably, a sharp increase in the number of samples with detectable *V. cholerae* O1 phages (57.6%) was observed around late September, coinciding with the peak *V. cholerae* O1 host detection in fecal specimens. Environmental water samples, analyzed between July and December, also revealed periodic fluctuations in *V. cholerae* O1 phages (Fig. 1E). The phage prevalence followed a seasonal trend, peaking multiple times between July and October before reducing toward the end of the year. Using culture-based approaches to detect *V. cholerae* O1 in environmental water specimens had been challenging in this study. However, from mid-August to September, when the cholera rate was the highest, 30%–40% water samples were found to be *V. cholerae* O1 positive in that period (Fig. S1).

We further analyzed the correlation between the weekly detection of *V. cholerae* O1 phages, isolated from both diarrheal specimens and wastewater samples, and *V. cholerae* O1-positive diarrheal cases. A statistically significant positive correlation was observed between the presence of *V. cholerae* O1 bacteria and *V. cholerae* phage in diarrheal stools (*r* = 0.53, *P* < 0.0001; Fig. 1C). Similarly, *V. cholerae* O1 phage detection in water samples also correlated significantly with the rate of cholera (*r* = 0.65, *P* = 0.0007; Fig. 1D).

**Fig 2:**
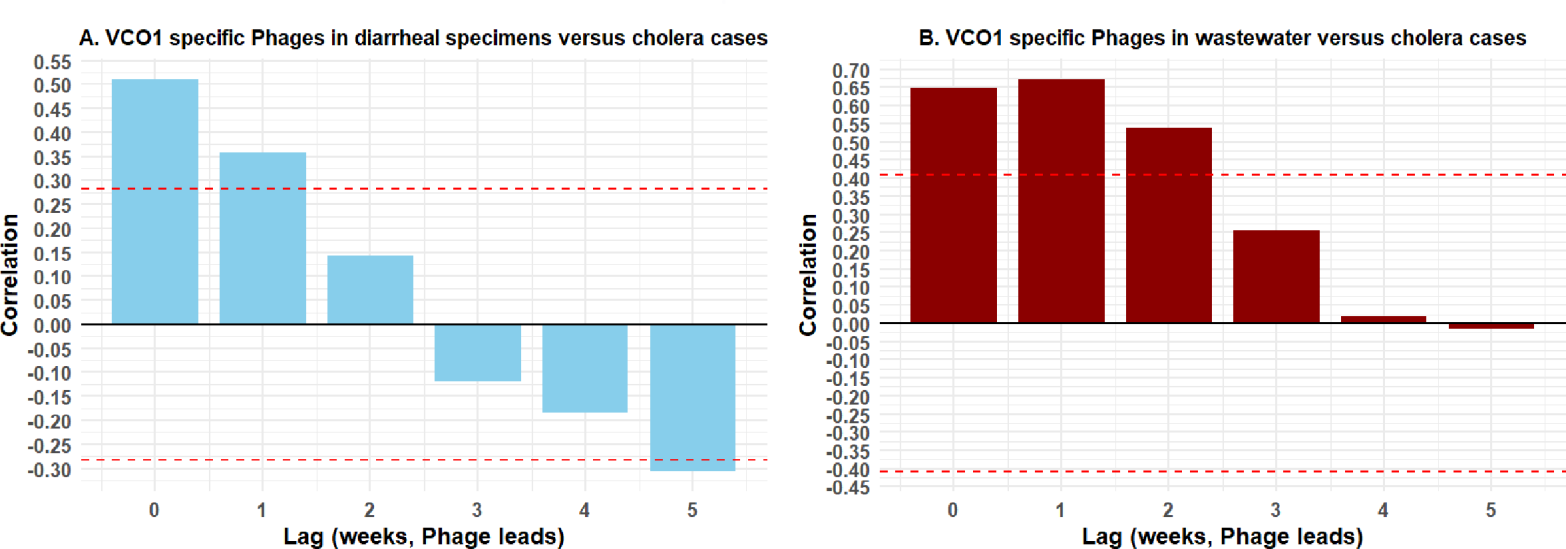
Time-lagged cross-correlation analyses of *V. cholerae* O1-specific phages and cholera cases. Correlation between the percentage of (**A**) *V. cholerae* O1 phages in diarrheal stool specimens and cholera cases and (**B**) *V. cholerae* O1 phages in environmental wastewater samples and cholera cases across a weekly time lag (phage leads). The dashed red lines represent the 95% confidence limits for statistical significance. Correlation values above or below these thresholds indicate significant associations at corresponding lag weeks.

We also performed a time-lagged correlation analysis to investigate the temporal association between the presence of *V. cholerae* O1-specific phages in stool or wastewater specimens and the proportion of cholera cases across weekly lags (Fig. 2). For stool-derived phages, the strongest correlation (r = 0.51) was found for co-occurrence of the presence of phages and proportion of cholera cases during the same week (Fig. 2A). Interestingly, for wastewater-derived phages, the highest correlation (*r* = 0.68) was found between phage detection in wastewater samples one week prior and an increased proportion of cholera cases in the following week (Fig. 2B). These findings suggest there is a potential application of vibriophages in cholera disease surveillance.

### Effect of rainfall and temperature on *V. cholerae* O1 and phages

**Fig 3:**
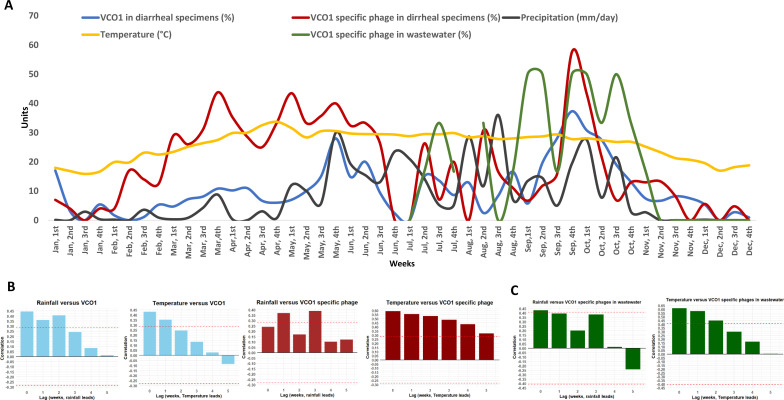
Association of rainfall and temperature with *Vibrio cholerae* O1 (VCO1) and phages from clinical and environmental sources. (**A**) Weekly rainfall (mm/day), temperature (°C), and isolation rates of VCO1 and phages from clinical specimens (January–December 2024) and environmental wastewater specimens (July–December 2024) are shown as different lines. (**B**) Cross-correlation analysis (**B**) between clinical VCO1 (blue bars) or phages (red bars) and rainfall or temperature across weekly time lags and (**C**) between environmental VCO1-specific phages (green bars) and rainfall or temperature across weekly time lags. The dashed red lines represent the 95% confidence limits for statistical significance. Correlation values above or below these thresholds indicate significant associations at corresponding lag weeks.

We evaluated the association of average weekly rainfall and temperature with the weekly percentage of *V. cholerae* O1 and corresponding phages from both clinical and environmental sources (Fig. 3). Cross-correlation analysis showed that rainfall and temperature on the same week and up to 1/2 preceding weeks were significantly associated with *V. cholerae* O1 (Fig. 3B) detection. In addition, rainfall in the preceding week was positively correlated with the rise of clinical phages in the following week (Fig. 3B and C). Similarly, increasing temperature showed a positive correlation with both *V. cholerae* O1 and phage detection in the same and subsequent weeks (Fig. 3B and C).

### Seasonal variation of ETEC and ETEC phages in clinical and environmental sources

**Fig 4:**
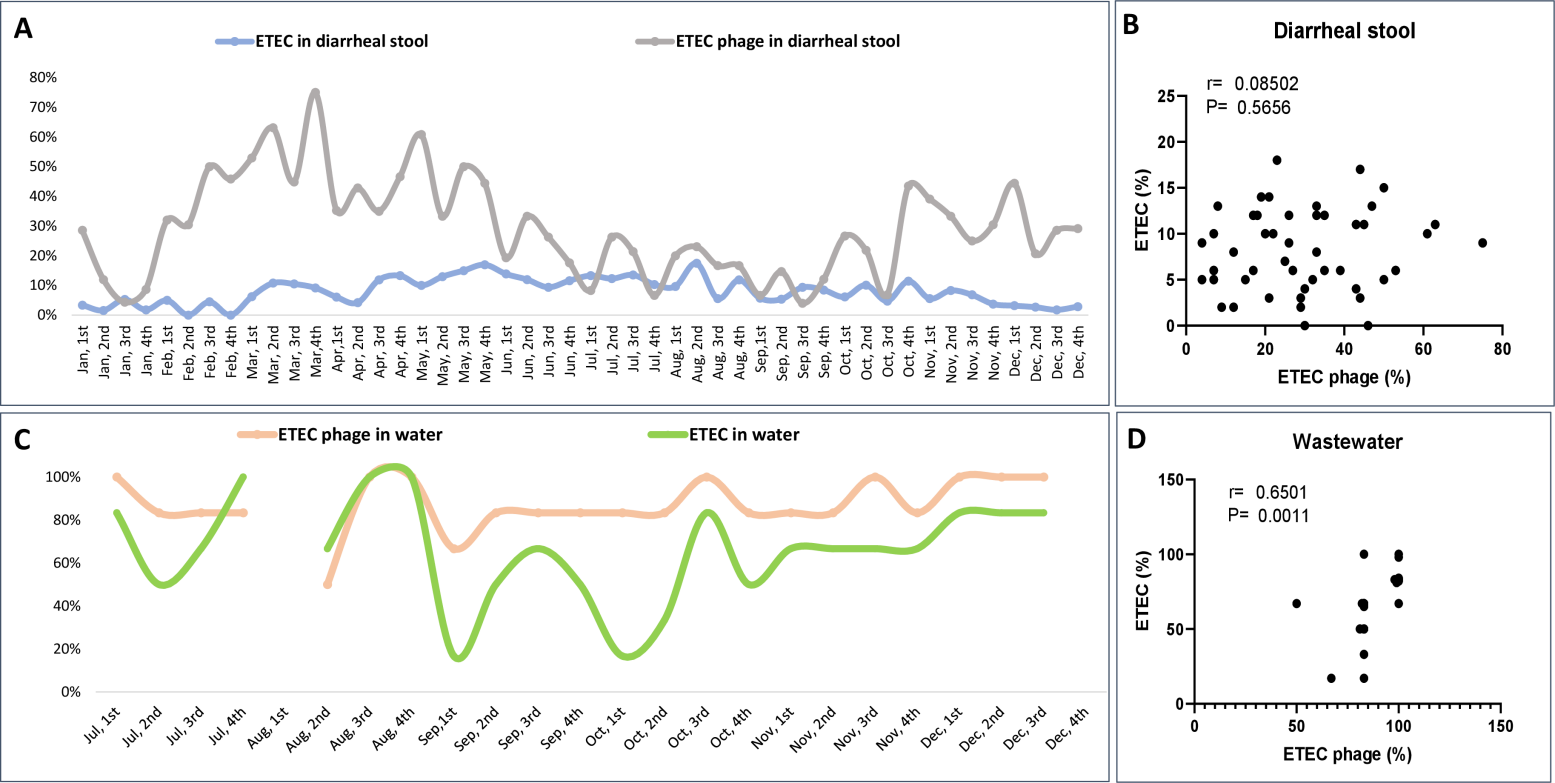
Temporal dynamics of ETEC and corresponding phages in clinical and environmental specimens. (**A**) Weekly isolation rate of ETEC and ETEC phages from diarrheal specimens collected between January and December 2024. (**B**) Correlation between the weekly percentage of ETEC and its phages isolated from diarrheal stools. (**C**) Weekly isolation rate of ETEC and ETEC phages from environmental wastewater specimens collected between July and December 2024. (**D**) Correlation between the weekly percentage of ETEC and its phages isolated from environmental wastewater specimens. Correlation analyses were performed using the Spearman test.

The temporal analysis of ETEC and corresponding phages in diarrheal stools showed the detection rate for ETEC in diarrheal stool fluctuated between 0% and 17%, whereas ETEC phage prevalence was higher, ranging between 4% and 75% (Fig. 4A). The ETEC phage showed periodic peaks, with the highest prevalence observed between March and May, followed by another increase towards the end of the year. Correlation analysis between ETEC and ETEC phage presence in fecal samples indicated no association (r = 0.08, Fig. 4B), suggesting that ETEC phage abundance in diarrheal samples may not directly reflect ETEC burden in infected individuals.

Similarly, ETEC and ETEC phages demonstrated substantial seasonal variation from environmental water samples between July and December (Fig. 4C). The prevalence of ETEC phages in water is consistently high, often exceeding 80%, whereas ETEC detection fluctuates significantly, with distinct declines in September and October. ETEC isolated from diarrheal stool did not follow a similar pattern, as such a high burden was found in ETEC from wastewater sources (*r* = −0.01; data not shown). However, there is a strong positive correlation between ETEC and ETEC phage abundance in water sources (Fig. 4D; *r* = 0.65, *P* < 0.001), indicating a significant relationship between bacterial and phage presence in the environment.

### Age-specific burden of diarrheal pathogens and phages

**Fig 5:**
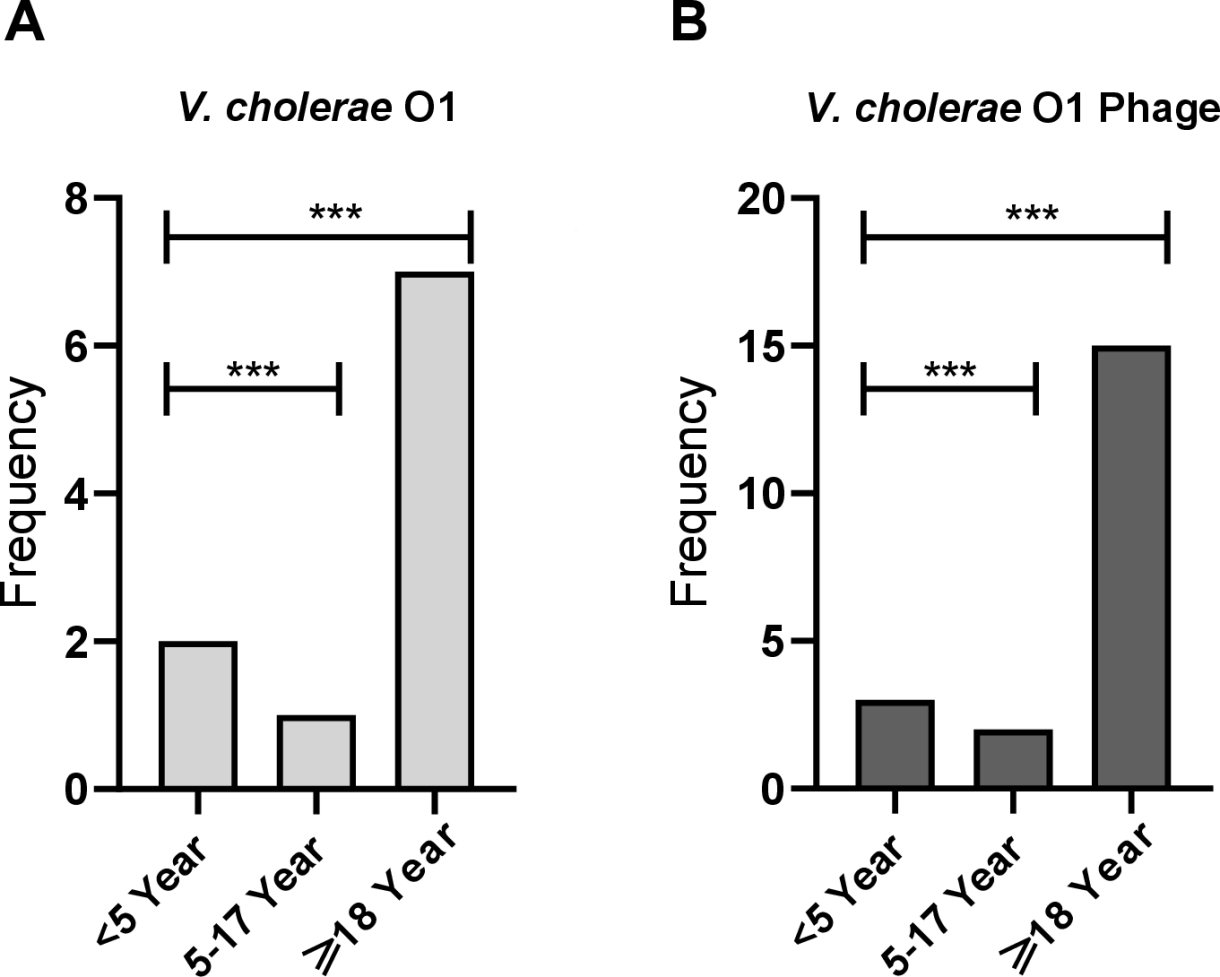
Age-specific burden of *Vibrio cholerae* O1 bacteria (**A**) and phages (**B**) in diarrheal specimens. For statistical analysis, χ^2^ tests were performed between the <5 year age group versus 5–17 year or ≥18 year groups. ***P < 0.0001.

Enteric infections with *V. cholerae*, ETEC, *Shigella*, and *Salmonella* exhibit age-specific distributions; for example, *V. cholerae* infections are more common in adults than children (5). We were interested in analyzing whether phages specific to these pathogens also follow similar age-related patterns. The distribution of *V. cholerae* O1 bacteria and phages among diarrheal cases was analyzed across three age groups: 0-5 years, >5–17 years, and ≥18 years (Fig. 5). Within all diarrheal patients studied, the presence of *V. cholerae* O1 was significantly higher among adults (≥18 years), with approximately 8% of diarrheal cases testing positive, compared with 2% in children aged 0–5 years and a slightly lower percentage in the 5–17 years group (*P* < 0.0001; Fig. 5A). A similar trend was observed for *V. cholerae* O1 phages, where their prevalence was markedly higher in the adult group (≥18 years), reaching nearly 18% of diarrheal cases, compared to around 4% in both the 0–5 years and >5–17 years groups (*P* < 0.0001; Fig. 5B). These findings suggest a significant age-dependent association between *V. cholerae* O1 and its associated phages, with the highest detection of both bacterial host and phages seen in adults. However, phages targeting ETEC, *Shigella*, and *Salmonella* spp. did not follow any age-specific pattern (data not shown).

### Cross-specificity of phages

**Fig 6:**
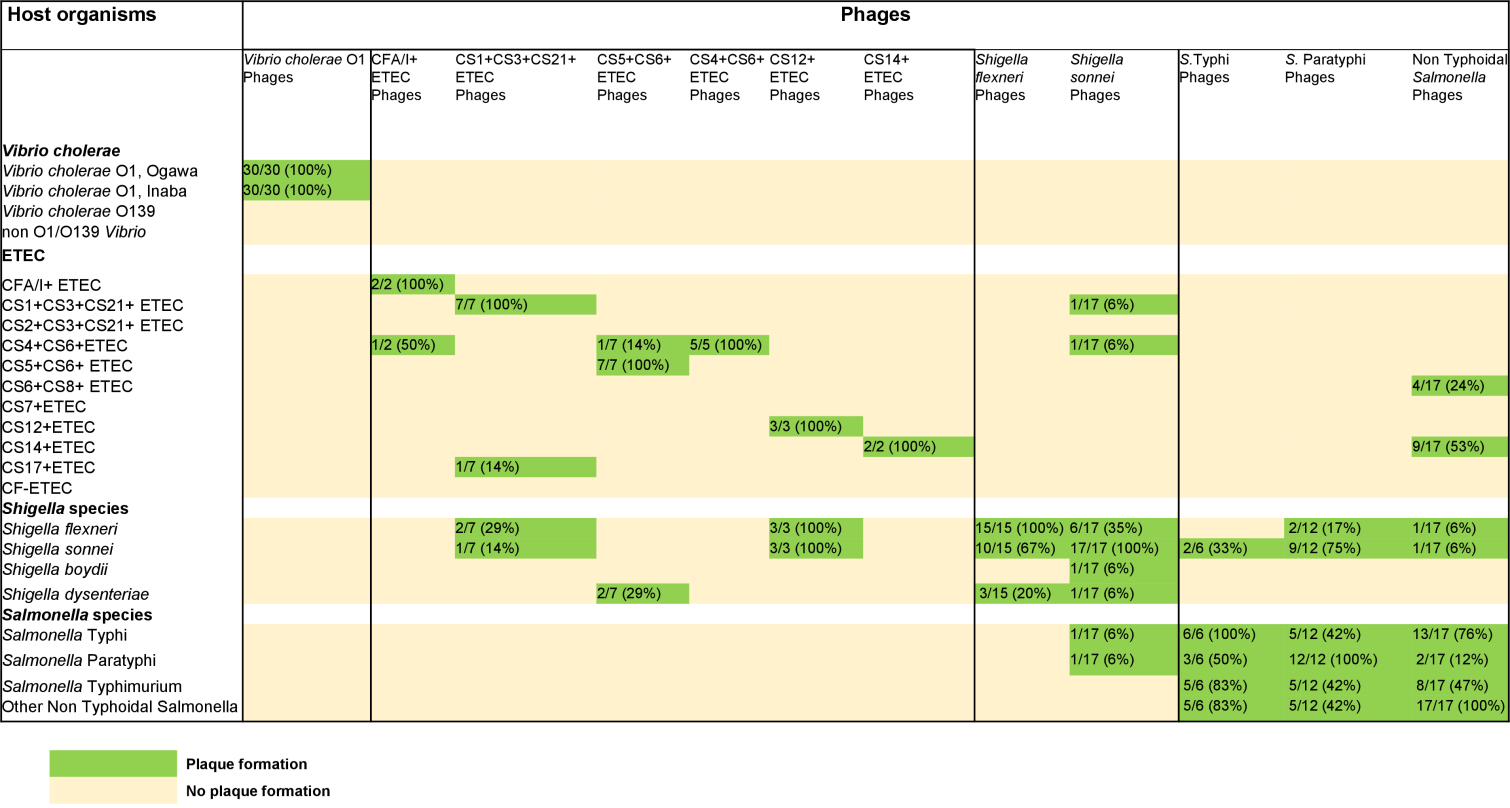
Host ranges of phages isolated against *Vibrio cholerae*, ETEC, *Shigella* spp., and *Salmonella* spp.

A subset of the purified phages isolated on one pathogen was tested for broad host ranges to 30 different bacterial host strains. *V. cholerae* O1 phages exhibited strict specificity for *V. cholerae* O1 and produced plaques on both the Ogawa and Inaba serotypes. However, *V. cholerae* O1 phages did not display cross-specificity toward *V. cholerae* O139, non-O1/O139 vibrios, or other bacterial species, including ETEC, *Salmonella* spp., and *Shigella* spp. (Fig. 6).

ETEC-specific phages demonstrated selective specificity for distinct colonization factor (CF)-positive ETEC strains. For example, phages targeting CS4+CS6+, CS14+, and CS12+ETEC strains failed to form plaques on other CF-positive ETEC strains. However, phages specific to CFA/I+, CS1+CS3+CS21+, and CS5+CS6+ETEC strains exhibited limited cross-infectivity with a subset of other CF-positive ETEC strains. Additionally, CS1+CS3+CS21+, CS5+CS6+, and CS12+ETEC phages displayed broad host ranges toward *Shigella flexneri*, *Shigella sonnei*, and *Shigella dysenteriae* but did not plaque on *V. cholerae* or *Salmonella* spp. (Fig. 5).

*Shigella flexneri* phages were capable of infecting all major serotypes, including *S. flexneri* 2a, 3a, and 6. However, these phages also exhibited cross-specificity toward *S. sonnei* and *S. dysenteriae*. A few phages isolated on *S. sonnei* showed a broad host range, plaquing on *S. flexneri*, *S. boydii, and S. dysenteriae* isolates as well as ETEC. One of the *S. sonnei* phages also plaqued on *S*. Typhi and *S*. Paratyphi hosts. None of the *Shigella* phages infected any tested strains of *V. cholerae*. Salmonella phages showed broad host specificity toward different species of *Salmonella*, *Shigella*, and ETEC (Fig. 5).

## DISCUSSION

Our study provides insights into the prevalence and dynamics of diarrheal pathogens and their corresponding bacteriophages in fecal and environmental wastewater specimens collected from Dhaka city, Bangladesh. To our knowledge, this is the first study to simultaneously investigate major diarrheal pathogens and their phages in both clinical and environmental sources longitudinally over time to assess the potential of bacteriophages as a tool to improve diarrheal disease surveillance. *V. cholerae* O1 phages are of particular interest and might emerge as a promising tool for cholera surveillance in both clinical and environmental settings. In Dhaka, Bangladesh, *V. cholerae* O1 and ETEC typically exhibit distinct biannual seasonal patterns, with two peaks occurring before and after the monsoon season. The first surge begins in the spring, at the onset of the hot season, whereas the second peak emerges in autumn, following the monsoons (20–23). Temporal analysis of *V. cholerae* O1 and its phages demonstrated significant seasonal alignment, with peak cholera prevalence occurring in late September, followed by an increase in *V. cholerae* O1-specific phages. A strong correlation between *V. cholerae* O1 phages and bacterial presence in both diarrheal stools and environmental water suggests a dynamic equilibrium, where phage abundance fluctuates in response to bacterial prevalence (23, 24). We also observed a strong correlation between the increased rate of wastewater phages in the preceding week and a rise in cholera cases in the following week, and vice versa. Our findings suggest that wastewater-derived *Vibrio* phages may serve as an early indicator for predicting cholera burden. A previous study has shown that environmental phages may influence the occurrence of epidemics and cholera seasonality (24). However, that study reported an inverse correlation between the environmental concentration of *Vibrio* phages and the presence of susceptible *V. cholerae* strains in the water samples collected from lakes or rivers. In contrast, our findings demonstrate a strong positive correlation, where an increase in cholera burden was associated with a rise in *V. cholerae* O1 phages in both clinical and wastewater samples collected from different sewage sources in Dhaka city. A moderate positive correlation was also observed for rainfall and temperature effect on the *V. cholerae* O1 and corresponding phages, which is consistent with earlier findings identifying rainfall as a driver for cholera seasonality (25).

In resource-poor settings, wastewater sampling, which commonly contains fecal matter, is an essential tool for public health surveillance, allowing the tracking of diseases caused by fecal-oral transmitted pathogens, as well as respiratory viruses such as SARS-CoV-2 (26, 27). Notably, we were unable to isolate culture-positive *V. cholerae* O1 from water samples throughout the year, except during the peak epidemic period. Due to limited resources, we were unable to test water samples by molecular techniques such as PCR, which could have detected *V. cholerae* O1 more sensitively (28). PCR-based detection requires a well-equipped laboratory and skilled personnel, making it less feasible for resource-limited field settings. Alternatively, given the minimal resources required for environmental phage surveillance, cholera phage detection using a simple plaque assay offers a practical and cost-effective approach. This method can be readily implemented in field settings across endemic regions like Bangladesh and can serve as a valuable complement to existing cholera surveillance strategies as well as to evaluate cholera vaccine effectiveness.

In addition to seasonal trends, we also observed age-specific correlations in cholera burden and phage abundance in stool. Age-stratified analysis revealed a significantly higher prevalence of *V. cholerae* O1 and its associated phages among adults (≥18 years) compared with younger age groups. This finding mirrors the established age-specific cholera burden observed in previous cholera surveillance studies in Bangladesh, which reported higher prevalence among adults than in younger populations (5, 29, 30). A similar trend was observed for *V. cholerae* O1 phages, suggesting that adults may be more frequently exposed to *V. cholerae* O1. Furthermore, the severity of diarrheal disease in cholera patients has been shown to be characterized by the phage-to-bacteria ratio in the human gut (31). All of these findings underscore the intricate interactions between bacterial pathogens and phages, highlighting their potential role in shaping disease epidemiology and transmission dynamics.

Phages targeting ETEC, *Shigella*, and *Salmonella* species were more frequently detected than their corresponding bacterial hosts in diarrheal stools, but did not follow seasonal patterns of variation like cholera phages in stools. By contrast, a significant positive correlation was observed between ETEC and its phages in water sources, implying that phage abundance may be influenced by bacterial availability in the aquatic environment. One possible explanation for the high abundance of these bacteriophages in stool and environmental water is their potential role in shaping bacterial populations, either by reducing pathogen loads in the human gut or by persisting in environmental reservoirs until favorable conditions arise for bacterial proliferation (Weinbauer, 2004; [6]. Our findings also suggest ETEC phages could be specific to colonization factors (CFs); phages specific to certain CF families (CFA/I+, CS1+CS3+CS21+, and CS6+CS8+ETEC) exhibited limited cross-infectivity with a subset of other CF-positive ETEC strains. These findings highlight the need for further investigation of these ETEC phages to find more prevalent ETEC CF-specific phages from the clinical and environmental settings.

Consistent with our findings, previous studies have also reported a high prevalence of *Shigella*-specific phages in various environmental samples, including wastewater (32, 33). Another possible factor contributing to the high abundance of ETEC- and *Shigella*-specific phages is the observed cross-specificity of certain phages, which enables them to infect both bacterial species. This adaptability may facilitate their persistence in the human gut by utilizing alternative hosts. Given that ETEC and *Shigella* share a common evolutionary lineage within the family *Enterobacteriaceae*, their phages may exhibit broad host ranges, a phenomenon that has been documented in different studies (32, 34). Our findings suggest that phages with a narrow host range show stronger correlations with the presence of their target pathogens, whereas those with a broader host range exhibit weak or no correlations. Despite the presence of resistance genes identified through genomic analysis of host bacterial strains, phages were able to infect and lyse these bacteria. This suggests that phages may possess adaptive mechanisms to overcome the bacterial defense systems. However, due to limited resources, we were unable to perform genomic sequencing of the phages to elucidate the underlying mechanisms of this evasion. Future studies should focus on elucidating the mechanisms underlying phage-host interactions, particularly the factors governing phage specificity and cross-infectivity.

There are several limitations to our study. Although we conducted weekly surveillance, data were collected for a single year, and environmental wastewater surveillance was limited to 6 months. This duration limits our ability to fully capture seasonal trends and highlights the need for longer, multi-year surveillance that simultaneously monitors both pathogens and phages in the same catchment area to validate these findings. Additionally, due to resource constraints, we used a culture-based method for the detection of *V. cholerae* in wastewater specimens instead of a more sensitive PCR-based method, which could have identified additional bacterial presence. Despite all these limitations, this study provides a strong background for future research involving wastewater and clinical samples, enabling more robust and multi-year surveillance of cholera in defined catchment areas.

This study highlights the intricate relationship between diarrheal pathogens and their corresponding phages, demonstrating significant seasonal variations, age-related patterns, and potential cross-infectivity. Longitudinal studies incorporating metagenomic analyses could provide deeper insights into the role of phages in bacterial evolution as well as antibiotic resistance dissemination. Overall, these exploratory findings suggest the possibility of integrating phage surveillance into diarrheal disease, particularly cholera, monitoring programs, and highlight the need for further large-scale and longitudinal research to validate the potential of phages as early-warning tools and surveillance markers for cholera.

## Data Availability

The whole genome sequence data of the host bacteria used to isolate phages have been submitted to the Sequence Read Archive (SRA) under the BioProject number PRJNA1295879. All the metadata linked to these strain numbers of each read-pair are available at http://www.ncbi.nlm.nih.gov/bioproject/1295879. Accession numbers of each read-pair sequence data are shown in Table S4.

## SUPPLEMENTAL MATERIAL

Supplemental material mbio.02654-25-s0001.docx Supplemental results, Fig. S1-S3, and Tables S1-S6.

ASM does not own the copyrights to Supplemental Material that may be linked to, or accessed through, an article. The authors have granted ASM a non-exclusive, world-wide license to publish the Supplemental Material files. Please contact the corresponding author directly for reuse.
