# Supplementary material for: Clinical and environmental wastewater-based bacteriophage surveillance for high-impact diarrheal diseases, including cholera, in Bangladesh
Source: mBio. 2025 Dec 9;17(1):e02654-25. doi: 10.1128/mbio.02654-25 (PMC12802145; doi:10.1128/mbio.02654-25)
Supplement: Supplemental material — Supplemental results, Fig. S1-S3, and Tables S1-S6. [file mbio.02654-25-s0001.docx]

**Supplementary Results**

**Phenotypic and genomic profile of host bacterial strains**

Using antibiogram assay (Supplementary Table 1), *V. cholerae* strains showed intermediate or full resistant to Erythromycin or Ampicillin. Most of the ETEC strains showed intermediate or full resistant to Azithromycin, Ceftriaxone, Erythromycin and Doxycycline. *Salmonella* spp. also showed intermediate resistant to Azithromycin and Ciprofloxacin. By whole genome sequence analysis, we also found genes responsible for these resistance for in the host isolates (Supplementary Table 2). For instance, Azithromycin resistance in ETEC was associated with mphA, Mrx (34, 35). Resistance to Nalidixic acid and intermediate resistance to ciprofloxacin in *Salmonella typhi* and *Salmonella* Paratyphi A were linked to gyrA_8, gyrA_9, gyrB_1, gyrB_3, parC_4, parE_2 mutation (36, 37). The presence of CTX-M and DHA-1 beta-lactamase genes in ETEC strains aligned with observed ceftriaxone resistance. Multidrug efflux pump genes were extensively identified across ETEC strains. Notably, discrepancies were observed where phenotypic Ampicillin intermediate resistance in *Vibrio cholerae* O1 lacked an identifiable beta-lactamase, suggesting intrinsic mechanisms. In addition to all these genes, numerous other resistance-associated genes were found in all host strains. We also found a diverse array of anti-phage defense systems in the *V. cholerae* O1, ETEC and *Salmonella* strains (Supplementary Figure 3). *V. cholerae* O1 *strains harbored Abi, BREX, CBASS, DISARM, Dnd, Gabija, Lamassu-Fam, Menshen, NixI, Retron, Restriction-Modification (RM) systems, Septu, ShosTA, TgvAB, Veles, Wadjet, WYL, and Zorya.* ETEC strains exhibited an even more extensive phage resistant genes, notably including multiple CRISPR-Cas systems (Type I-E, I-F, V-F), various Abi, BREX, CBASS, DISARM, Dnd, DRT, Druantia, Eleos, Gabija, Gao, JukAB, Lamassu-Fam, MADS, MazEF, Menshen, Mokosh, Paris, PD-Lambda, Retron, and diverse RM systems (Type I, II, III, IV). Salmonella strains also demonstrated comprehensive defense mechanisms, featuring BREX, CBASS, CRISPR-Cas (Type I-E, V-F), DISARM, Dnd, Dodola, Dpd, DRT, Druantia, Eleos, Erebus, Gabija, Gao, JukAB, Lamassu-Fam, Menshen, Mokosh, Paris, PD-Lambda, Pif, PrrC, Retron, RM systems (Type I, II, III), Septu, ShosTA, SspBCDE, Thoeris, Veles, Wadjet, WYL, and Zorya systems.

|  |  |  |  |  |  |  |  |  |  |  |  |  |  |
| --- | --- | --- | --- | --- | --- | --- | --- | --- | --- | --- | --- | --- | --- |
|  | Host organism | Azm | CIP | CRO | ERY | DOX | AMP | CFM | MEL | NA | SXT | CHL | AMC |
| 1 | *Vibrio cholerae* O1, ogawa | S | S | S | I | S | I | S | nt | nt | nt | nt | nt |
| 2 | *Vibrio cholerae* O1, inaba | S | S | S | I | S | I | S | nt | nt | nt | nt | nt |
| 3 | ETEC, CFA/I | S | S | S | R | I | nt | nt | S | nt | nt | nt | nt |
| 4 | ETEC, CS1+CS3+CS21 | R | S | I | R | I | nt | nt | S | nt | nt | nt | nt |
| 5 | ETEC, CS2+CS3+CS21 | R | S | S | R | S | nt | nt | I | nt | nt | nt | nt |
| 6 | ETEC, CS4+CS6 | R | S | R | R | S | nt | nt | S | nt | nt | nt | nt |
| 7 | ETEC, CS5+CS6 | S | S | S | I | I | nt | nt | S | nt | nt | nt | nt |
| 8 | ETEC, CS6+CS8 | R | S | S | R | I | nt | nt | S | nt | nt | nt | nt |
| 9 | ETEC, CS7 | R | S | S | R | I | nt | nt | S | nt | nt | nt | nt |
| 10 | ETEC, CS12 | S | S | R | I | I | nt | nt | S | nt | nt | nt | nt |
| 11 | ETEC, CS14 | S | S | I | R | I | nt | nt | S | nt | nt | nt | nt |
| 12 | ETEC, CS17 | R | S | S | R | R | nt | nt | S | nt | nt | nt | nt |
| 13 | ETEC, CF-LT+ | R | S | S | R | I | nt | nt | S | nt | nt | nt | nt |
| 14 | ETEC, CF-ST+ | S | S | I | R | S | nt | nt | S | nt | nt | nt | nt |
| 15 | *Salmonella* Typhi | S | I | S | nt | nt | S | S | nt | R | S | S | S |
| 16 | *Salmonella* Typhi | S | I | S | nt | nt | S | S | nt | R | S | S | S |
| 17 | *Salmonella* paratyphi A | I | I | S | nt | nt | S | S | nt | R | S | S | S |
| 18 | *Salmonella* group B | S | S | S | nt | nt | S | S | nt | S | S | S | S |
| 19 | *Salmonella* group C1 | S | S | S | nt | nt | S | S | nt | S | S | S | S |

**Supplementary Table 1. List of bacterial hosts and their antibiotic resistance patterns.**

**Abbreviations:** Azithromycin (AZM); Ciprofloxacin (CIP); Ceftriaxone (CRO); Erythromycin (ERY); Doxycycline (DOX); Ampicillin (AMP); Cefixime (CFM); Mecillinam (MEL); Nalidixic Acid (NA); Sulfamethoxazole (SXT); Chloramphenicol (CHL); Amoxicillin-clavulanic acid (AMC); Resistant (R); Intermediate resistant (I), Sensitive (S), not tested (nt).

**Supplementary Table 2. List of host bacterial antimicrobial resistance genes (ARGs).**

| **Antibiotic Class** | ***Vibrio cholerae* O1 ARG** | **Enterotoxigenic *E. coli* ARG** | ***Salmonella* ARG** |
| --- | --- | --- | --- |
| **Aminoglycosides** | APH_3____Ib, APH_6____Id, rrsB+ | AAC_6____Ib7, aadA-, kdpE, Pasteurella_multocida_16S | AAC_6___I-_3, AAC_6___I-3, rrsB+, Pasteurella_multocida_16S |
| **Beta-lactams** | - | Mycoplasma_-, Klebsiella_pneumoniae_PBP3, Klebsiella_pneumoniae_OmpK36, TEM-, CTX_M_-, DHA_1+, EC_13+ | Mycoplasma-, Klebsiella_pneumoniae_PBP3, Klebsiella_pneumoniae_OmpK36 |
| **Elfamycins** | Escherichia_coli_EF_Tu | Escherichia_coli_EF_Tu | Escherichia_coli_EF_Tu |
| **Fluoroquinolones** | Vibrio_cholerae_OmpT | gyrA_9, gyrB_1, parC_3, parE_1, QnrB-2, QnrS- | gyrA_8, gyrA_9, gyrB_1, gyrB_3, parC_4, parE_2 |
| **Macrolides** | Escherichia_coli_23S | Escherichia_coli_23S, mphA, Mrx, Moraxella_catarrhalis_23S | Escherichia_coli_23S, Moraxella_catarrhalis_23S |
| **Nitrofurantoins** | - | nfsA | - |
| **Peptide Antibiotics** | almE, almF, almG | bacA, eptA, mipA, PmrF, ugd, YojI | bacA |
| **Phenicols** | catB9, floR+ | - | - |
| **Phosphonic Acids** | - | Escherichia_coli_GlpT, Escherichia_coli_PtsI, Escherichia_coli_UhpT, murA_2, uhpA | Escherichia_coli_GlpT, Escherichia_coli_PtsI |
| **Rifamycins** | rpoB_2 | rpoB_1 | rpoB_1 |
| **Sulfonamides** | sul2 | folP_2, sul1 | folP_3 |
| **Tetracyclines** | rrsB+ | Escherichia_coli_LamB, rrsB+, tet_B_, tetR, | Escherichia_coli_LamB, rrsB+ |
| **Trimethoprims** | dfrA1 | dfrA1 | - |
| **Multidrug** | varG | acrA_2, acrB, acrD, AcrE, AcrF, AcrS, cpxA, CRP, cyaA_1, emrA, emrE_1, emrK, emrR, emrY, Escherichia_coli_AcrAB_TolC, Escherichia_coli_AcrAB_TolC_1, evgA, evgS, gadW, gadX, H_NS, marA, mdfA, mdtA, mdtB, mdtC, mdtE, mdtF, mdtG, mdtH, mdtM, mdtN, mdtO, mdtP, mlaD, mlaF, msbA ompF, porin_OmpC, soxR_1, soxS_1, TolC | acrB, CRP, cyaA_1, emrR, golS, H_NS, marA, mdsA, mdsB, mdsC, MdtK, msbA ramR, sdiA, soxR_2, soxS |
| **Others** | - | fabG, fabI, Klebsiella_pneumoniae_KpnH+, leuO, ompA, qacE- | fabG, fabI, OmpA |

**Supplementary Table 3. List of phage resistance genes in host bacterial strains.**

| **Bacterial strain** | **Anti-phage gene** |
| --- | --- |
| ***Vibrio cholerae* O1** | AbiEii__AbiEi_3, AbiEii__AbiEii, AbiJ__AbiJ, Belenos__VCA0457, Belisama__VCA0458, BREX__brxA_DUF1819, BREX__brxC, BREX__brxL, BREX__pglX1, BREX__pglX3, BREX__pglZA, BREX__pglZB, CBASS__AG_E1_ThiF, CBASS__AG_E2_Prok-E2_B, CBASS__Cyclase_II, CBASS__Jab, CBASS__Phospholipase, csf4_IV-A_1, csf4_IV-A1_1, dCTPdeaminase__dCTPdeaminase, DdmDE__DdmD, DdmDE__DdmE, DEDDh_I_II_III_IV_V_VI_1, Detocs__dtcC, dGTPase__Sp_dGTPase, DISARM__drmC, DISARM_2__drmMII, Divona__VCA0374, Dnd__DndA, Dodola__DolB, Dpd__FolE, Dpd__QueC, Dpd__QueD, Dpd__QueE, DRT2__RmuC, Druantia_II__DruM, DS-1__DS-1A, DS-23__DS-23, Epona__VCA0366, Esos__VCA0450, Gabija__GajB_1, Gabija__GajB_2, Gabija__GajB_3, Gao_Qat__QatD, GAPS2__GAPS2, Lamassu-Fam__LmuA_effector_Cap4_nuclease_II, Lamassu-Fam__LmuB_SMC_Cap4_nuclease_II, Lamassu-Fam__LmuB_SMC_Hydrolase_protease, Lamassu-Fam__LmuB_SMC_Lipase, Lamassu-Fam__LmuB_SMC_Mrr, Lamassu-Fam__LmuB_SMC_Sir2, Lamassu-Fam__LmuC_acc_Cap4_nuclease, Menshen__NsnA, Menshen__NsnB, Menshen__NsnC_2635923458, Menshen__NsnC_2726213746, Nantosuelta__VCA0322, Nemetona__VCA0441, NixI__NixI, NixI__Stix, Ogmios__VCA0308, Paris_II__AriA_AAA21, PD-Lambda-4__PD-Lambda-4_B, PfiAT__PfiA, PfiAT__PfiT, PrrC__EcoprrI, PsyrTA__PsyrA, PsyrTA__PsyrT, Retron__RT_Tot_1, Retron__RT_Tot_10, Retron__RT_Tot_11, Retron__RT_Tot_13, Retron__RT_Tot_14, Retron__RT_Tot_2, Retron__RT_Tot_3, Retron__RT_Tot_4, Retron__RT_Tot_5, Retron__RT_Tot_6, Retron__RT_Tot_7, Retron_I_A__ATPase_TypeIA, Retron_I_A__HNH_TIGR02646, Retron_VII_2__DUF3800, RM__Type_I_MTases_FAM_0, RM__Type_I_MTases_FAM_3, RM__Type_I_REases_FAM_0.einsi_trimmed, RM__Type_I_REases_FAM_1.einsi_trimmed, RM__Type_I_REases_FAM_2.einsi_trimmed, RM__Type_I_S_02, RM__Type_I_S_04, RM__Type_I_S_51, RM_Type_II__Type_II_MTases_FAM_0, RM_Type_II__Type_II_MTases_FAM_1, RM_Type_II__Type_II_MTases_FAM_15, RM_Type_II__Type_II_MTases_FAM_16, RM_Type_II__Type_II_MTases_FAM_17, RM_Type_II__Type_II_MTases_FAM_2, RM_Type_II__Type_II_MTases_FAM_23, RM_Type_II__Type_II_MTases_FAM_26, RM_Type_II__Type_II_MTases_FAM_3, RM_Type_II__Type_II_MTases_FAM_33, RM_Type_II__Type_II_MTases_FAM_4, RM_Type_II__Type_II_MTases_FAM_41, RM_Type_IIG__Type_IIG_4, RT_I_II_III_IV_V_VI_1, Septu__PtuA, Septu__PtuB, ShosTA__ShosA, ShosTA__ShosT, Sirona__VCA0356, Sucellos__SclA_VCA0367, Sucellos__SclB_VCA0368, TgvAB__TgvA, TgvAB__TgvB, Toutatis__TutB_VCA0447, UG35__UvrD, UG6__DnaG, UG9__DNA_pol, Veles__VlsB1, Veles__VlsB2, Veles__VlsC1, Veles__VlsC2, Wadjet__JetC_II, WYL_I_II_III_IV_V_VI_1, WYL_I_II_III_IV_V_VI_4, Zorya__ZorA, Zorya__ZorA2, Zorya__ZorB, Zorya_TypeI__ZorC, Zorya_TypeI__ZorD |
| **Enterotoxigenic *E. coli*** | Abi2__Abi_2, AbiA_small__AbiA_SLATT, AbiA_small__SLATT, AbiD__AbiD, AbiEii__AbiEi_3, AbiEii__AbiEi_4, AbiEii__AbiEii, AbiG__AbiGi, AbiH__AbiH, AbiJ__AbiJ, AbiP2__AbiP2, abc2, aca5, acric11, acriia7, arda, ardb, ardu, Avs_II__Avs2A, BREX__brxA_DUF1819, BREX__brxB_DUF1788, BREX__brxC, BREX__brxHII, BREX__brxL, BREX__pglX1, BREX__pglX3, BREX__pglZA, BREX__pglZB, Brig1__ADP_ribosyl, CBASS__2TM_Gros, CBASS__AG_E1_ThiF, CBASS__Cyclase_II, CBASS__Cyclase_SMODS, CBASS__Effector_4TM_S_4TM, CBASS__Sensing_SAVED, cas12f2_V-F_1, cas1_I-E_1, cas1_I-F_1, cas1_I_II_III_IV_V_VI_1, cas1_I_II_III_IV_V_VI_10, cas1_I_II_III_IV_V_VI_2, cas1_I_II_III_IV_V_VI_8, cas2_I-E_1, cas2_I-E_2, cas2_I_II_III_IV_V_VI_13, cas2_I_II_III_IV_V_VI_4, cas3HD_I_1, cas3_I_2, cas3_I_5, cas3f_I-F_1, cas5_I-E_13, cas5_I-E_2, cas5_I-E_3, cas5_I_9, cas5f_I-F_1, cas5f_I-F_2, cas5f_I-F_3, cas5f_I-F_4, cas6e_I_II_III_IV_V_VI_1, cas6e_I_II_III_IV_V_VI_2, cas6e_I_II_III_IV_V_VI_3, cas6f_I_II_III_IV_V_VI_1, cas6f_I_II_III_IV_V_VI_2, cas6f_I_II_III_IV_V_VI_3, cas7_I-E_16, cas7_I-E_2, cas7f_I-F_1, cas7f_I-F_2, cas8e_I-E_1, cas8e_I-E_2, cas8e_I-E_5, cas8f_I-F_3, cas8f_I-F_4, cas8f_I-F_8, cse2gr11_I-E_1, cse2gr11_I-E_2, cse2gr11_I-E_8, csf4_IV-A_1, dara, darb, DarTG__DarG, ddra, ddrb, DEDDh_I_II_III_IV_V_VI_1, DISARM__drmC, DISARM_2__drmMII, Dnd__DndA, Dnd__DndB, Dodola__DolB, Dpd__FolE, Dpd__QueC, Dpd__QueD, Dpd__QueE, DRT2__RmuC, DRT_3__SLATT, DRT_3__drt3a, DRT_3__drt3b, DRT9__SLATT, Druantia__DruE_3, Druantia_II__DruM, Druantia_III__DruH, DS-1__DS-1A, DS-15__DS-15A, DS-15__DS-15B, DS-15__DS-15C, DS-17__DS-17, DS-25__DS-25A, DS-25__DS-25B, DS-28__DS-28A, DS-28__DS-28B, DS-32__DS-32A, DS-32__DS-32B, DS-34__DS-34A, DS-36__DS-36, DS-37__DS-37, DS-9__DS-9B, Eleos__LeoA, Eleos__LeoA2, Eleos__LeoB, Eleos__LeoBC, Eleos__LeoC, FS_HEPN_TM__TM, Gabija__GajA, Gabija__GajB_1, Gabija__GajB_2, Gabija__GajB_3, gam, Gao_Her_SIR__HerA_SIR2, Gao_Her_SIR__SIR2, Gao_Hhe__HheA, Gao_Iet__IetA, Gao_Iet__IetS, Gao_Qat__QatD, Hachiman__HamA_1, Hachiman__HamB, hdf, JukAB__JukA, Kiwa__KwaA, Kiwa__KwaB, Kiwa__KwaB_2, klca, Lamassu-Fam__LmuA_effector_Sir2, Lamassu-Fam__LmuB_DS-30D, Lamassu-Fam__LmuB_SMC_Cap4_nuclease_II, Lamassu-Fam__LmuB_SMC_Hydrolase_protease, Lamassu-Fam__LmuB_SMC_Mrr, Lamassu-Fam__LmuC_DS-30C, lar, MADS__mad1, MazEF__MazE, MazEF__MazF, Menshen__NsnA, Menshen__NsnB, Menshen__NsnC_2507451963, Menshen__NsnC_2518623264, Menshen__NsnC_2583135648, Menshen__NsnC_2597471359, Menshen__NsnC_2601090942, Menshen__NsnC_2623244837, Menshen__NsnC_2632405575, Menshen__NsnC_2635923458, Menshen__NsnC_2656189766, Menshen__NsnC_2705968471, Menshen__NsnC_2723509649, Menshen__NsnC_2726213746, Menshen__NsnC_2726216653, MMB_gp29_gp30__gp29, Mok_Hok_Sok__Hok, Mok_Hok_Sok__Mok, Mokosh_type_I__MkoA_A, Mokosh_type_I__MkoA_B, Mokosh_type_I__MkoA_C, Mokosh_type_I__MkoA_D, Mokosh_type_I__MkoB_A, Mokosh_type_I__MkoB_C, Mokosh_TypeII__MkoC, narp1_adps, NLR_like_bNACHT01__NLR_like_bNACHT01, pAgo__GbbAgaS, pAgo__SIR2APAZ, Paris_I__AriA_AAA15, Paris_I__AriB_DUF4435_I, Paris_II__AriA_AAA21, Paris_II__AriB_DUF4435_II, PD-Lambda-1__PD-Lambda-1, PD-Lambda-2__PD-Lambda-2_C, PD-Lambda-4__PD-Lambda-4_A, PD-Lambda-4__PD-Lambda-4_B, PD-Lambda-5__PD-Lambda-5_B, PD-T4-3__PD-T4-3, PD-T4-5__PD-T4-5, PD-T7-2__PD-T7-2_A, PD-T7-2__PD-T7-2_B, PfiAT__PfiT, Pif__PifA, Pif__PifC, Prometheus__ProA, PrrC__EcoprrI, psia, psib, PsyrTA__PsyrA, PsyrTA__PsyrT, racc, Retron__RT_Tot_1, Retron__RT_Tot_10, Retron__RT_Tot_11, Retron__RT_Tot_12, Retron__RT_Tot_13, Retron__RT_Tot_14, Retron__RT_Tot_15, Retron__RT_Tot_4, Retron__RT_Tot_5, Retron__RT_Tot_6, Retron__RT_Tot_7, Retron__RT_Tot_8, Retron__RT_Tot_9, Retron_I_A__ATPase_TypeIA, Retron_I_A__HNH_TIGR02646, Retron_I_C__RT_1_C1, Retron_I_C__RT_1_C2, Retron_I_C__RT_1_C3, Retron_IX__HEPN, Retron_VI__HTH, Retron_VII_2__DUF3800, Retron_XI__RT_11, Retron_XII__RT_12, RloC__RloC, RM__Type_I_MTases_FAM_0, RM__Type_I_MTases_FAM_1, RM__Type_I_MTases_FAM_2, RM__Type_I_MTases_FAM_3, RM__Type_I_REases_FAM_0.einsi_trimmed, RM__Type_I_REases_FAM_1.einsi_trimmed, RM__Type_I_REases_FAM_2.einsi_trimmed, RM__Type_I_S_01, RM__Type_I_S_02, RM__Type_I_S_03, RM__Type_I_S_04, RM__Type_I_S_06, RM__Type_I_S_51, RM__Type_I_S_52, RM_Type_II__Type_II_MTases_FAM_0, RM_Type_II__Type_II_MTases_FAM_1, RM_Type_II__Type_II_MTases_FAM_10, RM_Type_II__Type_II_MTases_FAM_12, RM_Type_II__Type_II_MTases_FAM_13, RM_Type_II__Type_II_MTases_FAM_14, RM_Type_II__Type_II_MTases_FAM_15, RM_Type_II__Type_II_MTases_FAM_16, RM_Type_II__Type_II_MTases_FAM_17, RM_Type_II__Type_II_MTases_FAM_2, RM_Type_II__Type_II_MTases_FAM_21, RM_Type_II__Type_II_MTases_FAM_22, RM_Type_II__Type_II_MTases_FAM_23, RM_Type_II__Type_II_MTases_FAM_25, RM_Type_II__Type_II_MTases_FAM_26, RM_Type_II__Type_II_MTases_FAM_28, RM_Type_II__Type_II_MTases_FAM_29, RM_Type_II__Type_II_MTases_FAM_3, RM_Type_II__Type_II_MTases_FAM_30, RM_Type_II__Type_II_MTases_FAM_31, RM_Type_II__Type_II_MTases_FAM_32, RM_Type_II__Type_II_MTases_FAM_33, RM_Type_II__Type_II_MTases_FAM_36, RM_Type_II__Type_II_MTases_FAM_37, RM_Type_II__Type_II_MTases_FAM_4, RM_Type_II__Type_II_MTases_FAM_41, RM_Type_II__Type_II_MTases_FAM_6, RM_Type_II__Type_II_MTases_FAM_7, RM_Type_II__Type_II_MTases_FAM_8, RM_Type_II__Type_II_MTases_FAM_9, RM_Type_II__Type_II_REase02, RM_Type_II__Type_II_REase06, RM_Type_II__Type_II_REase13, RM_Type_II__Type_II_REase16, RM_Type_II__Type_II_REase19, RM_Type_II__Type_II_REase23, RM_Type_II__Type_II_REase27, RM_Type_II__Type_II_REase28, RM_Type_II__Type_II_REase30, RM_Type_II__Type_II_REase37, RM_Type_II__Type_II_REase38, RM_Type_IIG__Type_IIG_4, RM_Type_IIG__Type_IIG_FAM_0.einsi_trimmed, RM_Type_III__Type_III_MTases_FAM_0, RM_Type_III__Type_III_REases_FAM_0.einsi_trimmed, RM_Type_III__Type_III_REases_FAM_1.einsi_trimmed, RM_Type_IV__FAM_1, RM_Type_IV__FAM_2, RM_Type_IV__Type_IV_01, RM_Type_IV__Type_IV_05, RM_Type_IV__Type_IV_22, RosmerTA__RmrA_2585209417, RosmerTA__RmrA_2600853143, RosmerTA__RmrA_2603008502, RosmerTA__RmrA_2617826694, RosmerTA__RmrA_2623274509, RosmerTA__RmrA_2634882613, RosmerTA__RmrA_2634932349, RosmerTA__RmrA_2641389401, RosmerTA__RmrA_2662548665, RosmerTA__RmrA_2664250653, RosmerTA__RmrA_2676476075, RosmerTA__RmrA_2677172838, RosmerTA__RmrA_2734955840, RosmerTA__RmrA_2753529055, RosmerTA__RmrT_2585209417, Rst_Hydrolase-Tm__Hydrolase, Rst_Hydrolase-Tm__Hydrolase-Tm, Rst_RT-Tm__RT, Rst_RT-Tm__RT-Tm, Rst_RT-Tm__RT2, Rst_gop_beta_cll__beta, Rst_gop_beta_cll__cll, RT_I_II_III_IV_V_VI_1, SanaTA__SanaA, SanaTA__SanaT, SanaTA__SanaT_1, SDIC3__SDIC3C, SDIC3__SDIC3D, SDIC3__SDIC3E, SDIC3__SDIC3F, SDIC4__SDIC4B, Septu__PtuA, Septu__PtuA_2, Septu__PtuB, Septu__PtuB_2, Shango__SngC, ShosTA__ShosA, ShosTA__ShosT, SspBCDE__SspD, tad2, TgvAB__TgvA, TgvAB__TgvB, Thoeris__ThsB_Global, Thoeris_I__ThsA_new_grand, TIR-III__TIR-III_A, TIR-III__TIR-III_B, TniQ_I-F_1, UG17__UG17, UG35__UvrD, UG4__Fimbrae_114, UG4__Fimbrae_573, UG5_small__Nitrilase, UG6__DnaG, UG6__Nitrilase, UG9__DNA_pol, ulx, vcrx089, vcrx091, vcrx092, Veles__VlsB1, Veles__VlsB2, Veles__VlsC1, Veles__VlsC2, Wadjet__JetC_I, Wadjet__JetC_III, WYL_I_II_III_IV_V_VI_1, WYL_I_II_III_IV_V_VI_4, Zorya__ZorB |
| ***Salmonella*** | abc1, abc2, acric11, ardb, ardc, BREX__brxA_DUF1819, BREX__brxB_DUF1788, BREX__brxC, BREX__brxL, BREX__pglX1, BREX__pglX3, BREX__pglZA, BREX__pglZB, Brig1__ADP_ribosyl, CBASS__AG_E1_ThiF, cas12f2_V-F_1, cas1_I-E_1, cas1_I_II_III_IV_V_VI_2, cas1_I_II_III_IV_V_VI_8, cas2_I-E_1, cas2_I-E_2, cas2_I_II_III_IV_V_VI_4, cas3HD_I_1, cas3_I_2, cas3_I_5, cas5_I-E_13, cas5_I-E_2, cas5_I-E_3, cas5_I_9, cas6e_I_II_III_IV_V_VI_1, cas6e_I_II_III_IV_V_VI_2, cas6e_I_II_III_IV_V_VI_3, cas7_I-E_16, cas7_I-E_2, cas8e_I-E_1, cas8e_I-E_2, cas8e_I-E_5, cse2gr11_I-E_1, cse2gr11_I-E_2, cse2gr11_I-E_8, csf4_IV-A_1, DarTG__DarG, DEDDh_I_II_III_IV_V_VI_1, DISARM_2__drmMII, DISARM__drmC, Dnd_ABCDEFGH__DptF, Dnd_ABCDEFGH__DptG, Dnd_ABCDEFGH__DptH, Dnd__DndA, Dnd__DndB, Dnd__DndC, Dnd__DndD, Dnd__DndE, Dodola__DolB, Dpd__DpdB, Dpd__FolE, Dpd__QueC, Dpd__QueD, Dpd__QueE, DRT2__RmuC, Druantia__DruE_3, Druantia_II__DruM, Druantia_III__DruH, DS-1__DS-1A, DS-15__DS-15B, DS-15__DS-15C, DS-17__DS-17, DS-34__DS-34A, DS-9__DS-9A, DS-9__DS-9B, Eleos__LeoC, Erebus__EruA, Gabija__GajA, Gabija__GajB_1, Gabija__GajB_2, Gabija__GajB_3, gam, Gao_Iet__IetA, Gao_Qat__QatA, Gao_Qat__QatB, Gao_Qat__QatC, Gao_Qat__QatD, GAPS4__GAPS4a, JukAB__JukA, klca, Lamassu-Fam__LmuB_DS-30D, Lamassu-Fam__LmuB_SMC_Cap4_nuclease_II, Lamassu-Fam__LmuB_SMC_Hydrolase_protease, Lamassu-Fam__LmuC_DS-30C, Menshen__NsnB, Mokosh_TypeII__MkoC, Mokosh_type_I__MkoA_A, Mokosh_type_I__MkoA_D, Mokosh_type_I__MkoB_A, Mokosh_type_I__MkoB_B, Mokosh_type_I__MkoB_C, narp1_adps, Paris_I__AriA_AAA15, Paris_I__AriB_DUF4435_I, Paris_II__AriA_AAA21, Paris_II__AriB_DUF4435_II, PD-Lambda-4__PD-Lambda-4_B, PD-Lambda-5__PD-Lambda-5_B, PD-T4-1__PD-T4-1, PD-T4-3__PD-T4-3, PD_DExK_I_II_III_IV_V_VI_1, Pif__PifA, PrrC__EcoprrI, PsyrTA__PsyrA, PsyrTA__PsyrT, psia, psib, ral, Retron__RT_Tot_1, Retron__RT_Tot_10, Retron__RT_Tot_11, Retron__RT_Tot_12, Retron__RT_Tot_13, Retron__RT_Tot_14, Retron__RT_Tot_15, Retron__RT_Tot_2, Retron__RT_Tot_3, Retron__RT_Tot_4, Retron__RT_Tot_5, Retron__RT_Tot_6, Retron__RT_Tot_7, Retron__RT_Tot_8, Retron__RT_Tot_9, Retron_I_A__ATPase_TypeIA, Retron_I_A__HNH_TIGR02646, Retron_I_C__RT_1_C1, Retron_I_C__RT_1_C2, Retron_I_C__RT_1_C3, Retron_II__NDT, Retron_XI__RT_11, Retron_XII__RT_12, RM__Type_I_MTases_FAM_0, RM__Type_I_MTases_FAM_1, RM__Type_I_MTases_FAM_2, RM__Type_I_MTases_FAM_3, RM__Type_I_REases_FAM_0.einsi_trimmed, RM__Type_I_REases_FAM_1.einsi_trimmed, RM__Type_I_REases_FAM_2.einsi_trimmed, RM__Type_I_S_01, RM__Type_I_S_02, RM__Type_I_S_03, RM__Type_I_S_04, RM__Type_I_S_06, RM__Type_I_S_51, RM__Type_I_S_52, RM_Type_II__Type_II_MTases_FAM_0, RM_Type_II__Type_II_MTases_FAM_1, RM_Type_II__Type_II_MTases_FAM_12, RM_Type_II__Type_II_MTases_FAM_14, RM_Type_II__Type_II_MTases_FAM_15, RM_Type_II__Type_II_MTases_FAM_16, RM_Type_II__Type_II_MTases_FAM_2, RM_Type_II__Type_II_MTases_FAM_20, RM_Type_II__Type_II_MTases_FAM_21, RM_Type_II__Type_II_MTases_FAM_22, RM_Type_II__Type_II_MTases_FAM_23, RM_Type_II__Type_II_MTases_FAM_26, RM_Type_II__Type_II_MTases_FAM_28, RM_Type_II__Type_II_MTases_FAM_29, RM_Type_II__Type_II_MTases_FAM_3, RM_Type_II__Type_II_MTases_FAM_30, RM_Type_II__Type_II_MTases_FAM_32, RM_Type_II__Type_II_MTases_FAM_33, RM_Type_II__Type_II_MTases_FAM_37, RM_Type_II__Type_II_MTases_FAM_4, RM_Type_II__Type_II_MTases_FAM_8, RM_Type_II__Type_II_MTases_FAM_9, RM_Type_II__Type_II_REase06, RM_Type_II__Type_II_REase19, RM_Type_II__Type_II_REase22, RM_Type_II__Type_II_REase23, RM_Type_II__Type_II_REase27, RM_Type_II__Type_II_REase30, RM_Type_II__Type_II_REase35, RM_Type_IIG__Type_IIG_4, RM_Type_IIG__Type_IIG_FAM_0.einsi_trimmed, RM_Type_III__Type_III_MTases_FAM_0, RM_Type_III__Type_III_REases_FAM_0.einsi_trimmed, RM_Type_III__Type_III_REases_FAM_1.einsi_trimmed, Rst_3HP__Hp1, Rst_3HP__Hp2, Rst_3HP__Hp3, Rst_Hydrolase-Tm__Hydrolase, RT_I_II_III_IV_V_VI_1, SDIC3__SDIC3C, SDIC3__SDIC3D, SDIC3__SDIC3E, SDIC3__SDIC3F, SDIC4__SDIC4A, Septu__PtuA, Septu__PtuB, ShosTA__ShosA, SspBCDE__SspD, Thoeris__ThsB_Global, UG35__UvrD, UG4__Fimbrae_114, UG4__Fimbrae_573, UG4__Fimbrae_972, UG6__DnaG, UG9__DNA_pol, vcrx091, vcrx092, Veles__VlsB1, Veles__VlsB2, Veles__VlsC1, Veles__VlsC2, Wadjet__JetC_I, Wadjet__JetC_II, Wadjet__JetC_III, WYL_I_II_III_IV_V_VI_1, WYL_I_II_III_IV_V_VI_4, Zorya__ZorA2, Zorya__ZorB |

**Supplementary Table 4. Biosample accession numbers of host bacterial whole genome sequence data.**

| **Isolate** | **Accession numbers** |
| --- | --- |
| *Vibrio cholerae* O1, Ogawa | SAMN50191339 |
| *Vibrio cholerae* O1, Inaba | SAMN50191340 |
| ETEC (CFA/I+CS21) | SAMN50191341 |
| ETEC (CS1+CS3+CS21) | SAMN50191342 |
| ETEC (CS2+CS3+CS21) | SAMN50191343 |
| ETEC (CS4+CS6) | SAMN50191344 |
| ETEC (CS5+CS6) | SAMN50191345 |
| ETEC (CS7) | SAMN50191346 |
| ETEC (CS6+ CS8) | SAMN50191347 |
| ETEC (CS12) | SAMN50191348 |
| ETEC (CS14) | SAMN50191349 |
| ETEC (CS17) | SAMN50191350 |
| ETEC (CF-ve, LT+ve) | SAMN50191351 |
| ETEC (CF-ve, ST+ve) | SAMN50191352 |
| *Salmonella enterica* Typhi | SAMN50191353 |
| *Salmonella* *enterica* Typhi | SAMN50191354 |
| *Salmonella* *enterica* Paratyphi A | SAMN50191355 |
| *Salmonella* *enterica* group B | SAMN50191356 |
| *Salmonella* *enterica* group C1 | SAMN50191357 |

**Supplementary Table 5.** **Isolation rate of *V. cholerae* O1, ETEC and *Shigella* bacteria and corresponding phages isolated from environmental wastewater specimens.**

|  | **Bacterial pathogens,**  **n (%)** | | **Phages,**  **n (%)** |
| --- | --- | --- | --- |
| **Number of tested specimens** | 144 | 144 | |
| *V. cholerae* O1 | 6 | 28 | |
| ETEC | 91 | 121 | |
| *Shigella* spp. | 0 | 138 | |
| *Salmonella* spp. | 2 | 128 | |

**Supplementary Table 6. Weekly counts of diarrheal pathogens and phages from diarrheal specimens in year 2024.**

| Weeks | Pathogen (count) | | | | | Phages (count) | | | | |
| --- | --- | --- | --- | --- | --- | --- | --- | --- | --- | --- |
|  | Tested specimens | VCO1^a^ | ^b^ETEC | *Shigella* spp. | *Salmonella* spp. | Tested specimens | VCO1 phage | ETEC phage | Shigella phage | Salmonella phage |
| Jan 1st | 59 | 1 | 2 | 0 | 0 | 14 | 1 | 4 | nd | nd^c^ |
| Jan 2nd | 64 | 2 | 1 | 0 | 2 | 25 | 1 | 3 | nd | nd |
| Jan 3rd | 56 | 0 | 3 | 2 | 2 | 23 | 0 | 1 | nd | nd |
| Jan 4th | 56 | 3 | 1 | 1 | 0 | 23 | 1 | 2 | nd | nd |
| Feb 1st | 60 | 1 | 3 | 0 | 1 | 24 | 1 | 8 | nd | nd |
| Feb 2nd | 78 | 0 | 0 | 1 | 0 | 24 | 4 | 7 | nd | nd |
| Feb 3rd | 88 | 2 | 4 | 0 | 0 | 22 | 3 | 11 | 3 | nd |
| Feb 4th | 57 | 3 | 0 | 3 | 1 | 24 | 3 | 11 | 17 | nd |
| Mar 1st | 64 | 3 | 4 | 1 | 0 | 17 | 5 | 9 | 8 | nd |
| Mar 2nd | 83 | 6 | 9 | 1 | 0 | 19 | 4 | 12 | 14 | nd |
| Mar 3rd | 86 | 7 | 9 | 1 | 5 | 29 | 9 | 13 | 16 | nd |
| Mar 4th | 65 | 7 | 6 | 0 | 0 | 29 | 10 | 20 | 22 | nd |
| Apr 1st | 49 | 5 | 3 | 0 | 2 | 17 | 6 | 6 | 14 | nd |
| Apr 2nd | 47 | 5 | 2 | 0 | 0 | 14 | 4 | 6 | 11 | nd |
| Apr 3rd | 59 | 4 | 7 | 1 | 3 | 20 | 5 | 7 | 14 | nd |
| Apr 4th | 60 | 4 | 8 | 3 | 0 | 15 | 5 | 7 | 8 | nd |
| May 1st | 59 | 4 | 6 | 0 | 1 | 23 | 10 | 14 | 17 | nd |
| May 2nd | 67 | 7 | 9 | 0 | 0 | 24 | 8 | 8 | 15 | nd |
| May 3rd | 68 | 10 | 10 | 4 | 2 | 28 | 12 | 14 | 26 | nd |
| May 4th | 87 | 24 | 15 | 5 | 2 | 45 | 20 | 20 | 34 | nd |
| Jun 1st | 108 | 16 | 15 | 2 | 3 | 31 | 11 | 6 | 20 | 2 |
| Jun 2nd | 75 | 15 | 9 | 1 | 1 | 27 | 9 | 9 | 18 | 4 |
| Jun 3rd | 53 | 5 | 5 | 0 | 2 | 19 | 5 | 5 | 13 | 8 |
| Jun 4th | 43 | 1 | 5 | 0 | 5 | 17 | 0 | 4 | 13 | 3 |
| Jul 1st | 45 | 0 | 6 | 2 | 4 | 12 | 0 | 10 | 7 | 2 |
| Jul 2nd | 65 | 10 | 8 | 0 | 5 | 19 | 3 | 5 | 11 | 0 |
| Jul 3rd | 59 | 8 | 8 | 2 | 4 | 14 | 1 | 3 | 7 | 0 |
| Jul 4th | 58 | 5 | 6 | 2 | 3 | 15 | 3 | 1 | 8 | 0 |
| Aug 1st | 31 | 4 | 2 | 0 | 1 | 15 | 0 | 3 | 8 | 0 |
| Aug 2nd | 40 | 1 | 7 | 0 | 0 | 13 | 4 | 3 | 5 | 1 |
| Aug 3rd | 36 | 3 | 2 | 0 | 0 | 12 | 2 | 2 | 6 | 1 |
| Aug 4th | 42 | 7 | 5 | 2 | 0 | 18 | 2 | 3 | 7 | 0 |
| Sep 1st | 35 | 2 | 2 | 1 | 1 | 15 | 1 | 1 | 4 | 1 |
| Sep 2nd | 56 | 11 | 3 | 2 | 2 | 34 | 4 | 5 | 11 | 2 |
| Sep 3rd | 64 | 18 | 6 | 1 | 2 | 30 | 5 | 1 | 9 | 0 |
| Sep 4th | 59 | 22 | 5 | 2 | 1 | 28 | 18 | 4 | 12 | 6 |
| Oct 1st | 65 | 20 | 4 | 0 | 1 | 30 | 13 | 8 | 16 | 3 |
| Oct 2nd | 69 | 19 | 7 | 1 | 1 | 30 | 6 | 7 | 12 | 2 |
| Oct 3rd | 63 | 12 | 3 | 0 | 3 | 31 | 3 | 2 | 10 | 0 |
| Oct 4th | 61 | 8 | 7 | 0 | 5 | 27 | 5 | 10 | 11 | 0 |
| Nov 1st | 54 | 4 | 3 | 2 | 4 | 23 | 3 | 9 | 14 | 7 |
| Nov 2nd | 60 | 4 | 5 | 1 | 1 | 15 | 3 | 5 | 7 | 0 |
| Nov 3rd | 73 | 6 | 5 | 1 | 3 | 19 | 0 | 6 | 9 | 2 |
| Nov 4th | 80 | 6 | 3 | 0 | 2 | 23 | 0 | 7 | 9 | 7 |
| Dec 1st | 93 | 5 | 3 | 2 | 0 | 18 | 1 | 8 | 11 | 2 |
| Dec 2nd | 110 | 0 | 3 | 1 | 0 | 29 | 0 | 6 | 8 | 0 |
| Dec 3rd | 109 | 3 | 2 | 3 | 0 | 21 | 1 | 6 | 9 | 2 |
| Dec 4th | 105 | 1 | 3 | 1 | 1 | 24 | 0 | 7 | 9 | 0 |

^a^*Vibrio cholerae* O1 (VCO1)

^b^Enterotoxigenic *E. coli*

^c^Not done (nd)

**Supplementary Figures**

**Supplementary Figure 1: Weekly isolation of VCO1 from wastewater specimens from July-December, 2024.**

**Supplementary Figure 2. Temporal dynamics of Shigella and Salmonella and corresponding phages in clinical specimens.** (A) Weekly isolation rate of Shigella and Shigella phages and (B) of Salmonella and its phages from diarrheal specimens collected between July to December 2024.

**Supplementary Figure 3. Phage dose responses effect on (A) plaque forfation and (B) *V. cholerae* O1 growth.**
